# The ANTI-LAMBDA: A Non-statical Tonal Indicator Low-Frequency Air-Bone Gap of Ménière’s Bouts and Disease Activity

**DOI:** 10.7759/cureus.66249

**Published:** 2024-08-05

**Authors:** Francisco Alves de Sousa, João Tarrio, Joana Ida Dias, Ana Pinto, Luís Meireles, Ângela Reis Rego

**Affiliations:** 1 Otolaryngology - Head and Neck Surgery, Unidade Local de Saúde de Santo António, Porto, PRT; 2 Neuroradiology, Unidade Local de Saúde de Santo António, Porto, PRT; 3 Otolaryngology - Head and Neck Surgery, Instituto CUF Porto, Porto, PRT; 4 Otolaryngology - Head and Neck Surgery, Hospital de Santa Maria, Porto, PRT

**Keywords:** novel marker, low-frequency thresholds, vertigo diagnosis, disease stability, audiometry, anti-lambda, attacks, hydrops, air-bone gap, ménière

## Abstract

Introduction: There have been reports of patients with Ménière´s disease (MD) showing unexplained audiometric air-bone gaps at low frequencies. Little is known about the clinical significance of this finding. The objective of this study was to describe this phenomenon while relating it with clinical features, namely the incidence of attacks.

Methods: Unilateral MD patients were selected and cerebral magnetic resonance imaging (cMRI) was assessed to exclude structural pathology. A retrospective longitudinal analysis regarding disease activity and audiometric details was performed. A coincidence index and regression predictive models were considered to assess the relationship between the air-bone gap and MD activity.

Results: A total of 70 MD patients were enrolled and 252 audiograms were assessed. Low-frequency air-bone gaps (LFABGs) were significantly associated with unstable MD (p < 0.001), demonstrating a sensitivity and specificity of 93.8% and 82.7%, respectively. The incidence of unstable disease with the presence of LFABG was 89.1 %. A higher LFABG magnitude correlated with increased disease activity (p < 0.001) and was particularly pronounced at 250 Hz and 500 Hz.

Conclusion: The typical LFABG encountered was here called ANTI-LAMBDA (A Non-statical Tonal Indicator Low-Frequency Air-Bone Gap of Ménière’s Bouts and Disease Activity). It relates to MD activity and is a potential new tool to assess MD stability/need for additional therapeutics.

## Introduction

Ménière’s disease (MD) is a multifactorial inner ear disorder characterized by episodic vestibular symptoms, fluctuating hearing loss, tinnitus, and aural fullness [1,2]. As no objective methods exist for diagnosis, the Committee on Hearing and Equilibrium of the American Academy of Otolaryngology-Head & Neck Surgery (AAO-HNS) proposes the use of symptom-based guidelines for the diagnosis of MD [3], which has been endorsed by the Barany Society [4]. Endolymphatic hydrops (EH) are a histological feature of MD [5]. While all MD patients have EH, not all EH patients experience MD symptoms [6,7]. MD is histologically characterized by dilation of the cochlear scala media and displacement of Reissner’s membrane into the vestibular scala [8,9]. Saccular findings are also prevalent, whereas utricular EH are rarely observed [9].

The pattern of hearing loss most characteristic of MD is fluctuating low- to medium-frequency sensorineural hearing loss (SNHL) [4]. An already described but less visited form of hearing loss is the phenomena of low-frequency air-bone gaps (LFABGs) in MD. They occur in the absence of middle ear disease and are an unexplored topic in MD [10]. Intriguingly, LFABGs have been shown to occur in up to 33% of MD patients [10-13]. Studies using novel MD imaging sequences such as the one of Sugimoto et al. [14] and Pai et al. [10] suggest that the appearance of LFABGs in the context of chronic EH suggests aggravation. It is suggested that increased perilymphatic pressure dampens stapedial motion or that saccule dilatation applies direct stress to the stapes footplate, halting sound conduction [13,14]. Could this be true, LFABGs would be a useful indicator for evaluating and treating patients with MD, as they could alert the clinician about the inner ear status. Following the identification of LFABGs, magnetic resonance imaging (MRI) with hydrops protocol could further confirm the presence of EH and guide clinicians in determining the most suitable measures. Ultimately, clinical valorization of LFABGs could help prevent disabling attacks by supporting therapeutic actions toward preventive stabilization.

To date, there have been few reports on the incidence and prevalence of LFABGs in MD [11-13], and there is limited information about the clinical significance of LFABGs [13]. LFABGs in MD constitute a fairly unknown topic for general otolaryngologists. On the other hand, they are a quite unique feature from the otologic perspective, as they have been shown to be fluctuant or transient within the same MD patient [13]. In this regard, the objective of the present study was to properly describe LFABGs in MD in terms of audiometric components, while relating it to Ménière´s disease activity (MDA). We here ultimately describe a new audiometric pattern for MD´s LFABGs: the ANTI-LAMBDA (a Non-statical Tonal Indicator Low-Frequency Air-Bone Gap of Ménière’s Bouts and Disease Activity).

## Materials and methods

Study design, settings, and participants

In order to carry out an observational retrospective cohort study, clinical information and audiological profiles from patients with MD were reviewed. Data was retrieved from the clinical records of patients followed between 2013 and 2023. The information was collected by a single investigator, followed by a second-look confirmation of the selected sample by the senior author.

Inclusion criteria were unilateral definite MD according to the joint consortium of Bárany Society, EAONO, the AAO-HNS, the Japan Society for Equilibrium Research, and the Korean Balance Society (i.e., observation of an episodic vertigo condition accompanied by low-to-medium-frequency SNHL and fluctuating aural symptoms (hearing, tinnitus, and/or fullness) in the afflicted ear + length of vertigo episodes restricted to 20 minutes to 12 hours) [4]; age ≥ 18 years; available cerebral magnetic resonance imaging (MRI); absence of other symptomatic relatable lesions on MRI.

**Figure 1:**
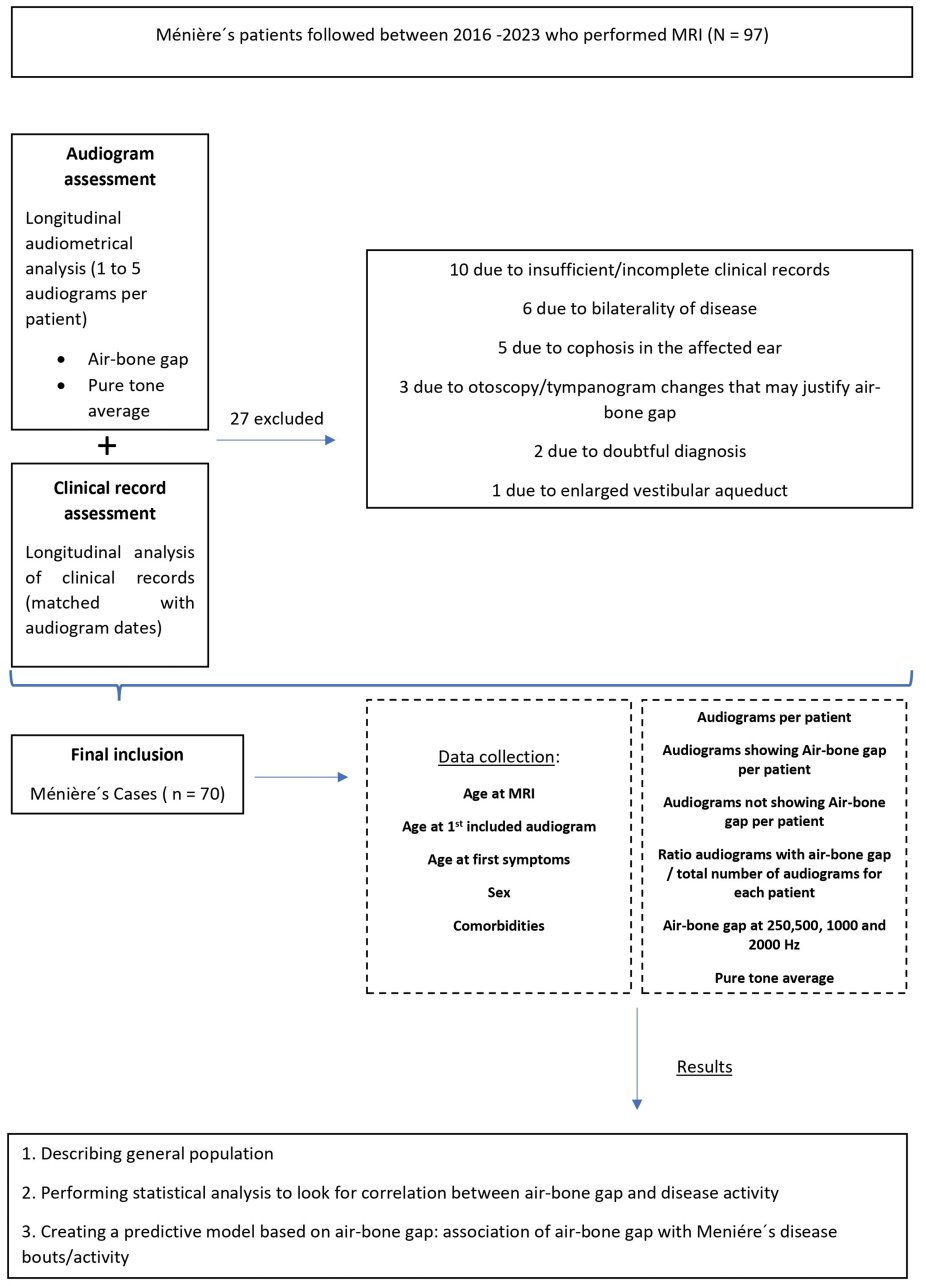
Methodological approach flow chart

Exclusion criteria were bilateral MD; third window syndrome (example: enlarged vestibular aqueduct or canal dehiscence, confirmed with additional thin sliced tomography with Polsh incidence); altered otoscopy and/or impedance audiometry (tympanometry) raising suspicion of middle ear alterations (example: eardrum perforation, myringosclerosis, eardrum retraction); cophosis in the MD's afflicted ear; absence of available cerebral MRI and inexistent; unavailable or insufficient audiological data. Figure 1 depicts the overall methodological approach.

Instruments, variables, and data collection

Data collection was conducted through a systematic, multi-stage process, wherein audiometric, imaging, clinical, and demographic data were documented and consolidated within a unified database. 

Audiometric Findings

The initial phase of data collection focused on audiological parameters, including LFABG values for each frequency across 250 Hz, 500 Hz, 1000 Hz, and 2000 Hz, and the pure-tone average (PTA). To standardize data collection across patients with extended follow-up durations, only the first five available audiograms (AUDs) were included, resulting in a range of one to five AUD per participant. The date of each AUD assessment was also recorded. Pure-tone audiometry was performed according to standard clinical protocols, with appropriate masking of the contralateral ear employed whenever necessary to eliminate the possibility of cross-hearing and to ensure the accurate identification and confirmation of the LFABG.

Imagiological Findings

Brain magnetic resonance imaging (MRI) was assessed in all cases to exclude alternative diagnoses, such as cerebellopontine angle tumors or other inner ear pathologies. All MRI scans were evaluated by a neuroradiologist to ensure the consistency and accuracy of the assessment.

Clinical Findings

Clinical information documented by the attending otolaryngologist on the day of each AUD was retrieved. MDA was classified as either "stable" or "unstable" based on a comprehensive review of the clinical records. Disease stability was defined as the absence of reported aural symptoms, hearing loss, or vertigo, and the absence of any need to adjust medication within the three months preceding the AUD. Conversely, disease instability was defined when the patient reported any of these symptoms or required medication adjustments to maintain stability.

Demographic Findings

Additional pertinent variables were extracted from clinical records, including age, sex, and the presence of comorbid conditions.

Statistical methods

IBM SPSS Statistics for Windows, Version 29 (Released 2023; IBM Corp., Armonk, New York, United States) was used for statistical analysis. Categorical variables were reported as percentages in the descriptive analysis, whereas continuous variables were shown as means and standard deviations after confirming normal distribution through skewness, kurtosis, and the Kolmogorov-Smirnov test. Correlations were analyzed using the chi-square test for categorical variables and the Spearman´s test for continuous ones. In order to adjust for potential confounders and obtain a model of risk of MD activity based on LFAGs, a linear regression analysis by mixed ANOVA was performed to increase statistical validity. All p-values presented are two-tailed, with a p-value ≤ 0.05 indicating statistical significance.

Ethics

This study was conducted in accordance with the Declaration of Helsinki and received approval from the Institutional Ethics Committee (approval number: 2024.048(044-DEFI/044-CE)). Informed consent was waived due to the retrospective nature of the study and the anonymized use of patient data.

## Results

Study population

In this study, a total of 97 patients were initially considered for inclusion. Following the application of the eligibility criteria, the final cohort comprised 70 individuals (Figure 1). The number of AUDs with corresponding adequate clinical information varied among participants. Twenty-four patients (34.3%) underwent five AUDs, 16 (22.9%) had four AUDs, 13 (18.6%) had three AUDs, 12 (17.1%) had two AUDs, and five (7.1%) completed only one AUD with matching clinical data. In total, 252 AUDs were assessed and matched with clinical information on disease. Other relevant sample characteristics are displayed in Table 1.

**Table 1:** Population characteristics *Pure tone average (PTA) was calculated as the three-tone average of 0.5, 1, and 2 KHz. † Product of 252 entries (total number of collected audiograms); Ꝭ includes asthma; ‡ includes depression

Continuous variables	Mean (± standard deviation)	Categorical variables	Number of cases ((Frequency (%))
Patients (n = 70)			
Age at first symptoms (years) ^1^	41.2± 11.6	Sex (female)	42 (60)
		Comorbidities	
		Diabetes Mellitus	13 (18.6)
		Hypertension	24 (34.3)
Time from first symptoms to MRI (years)	6.5± 8.1	Smoking	8 (11.4)
		Obesity	6 (8.6)
Time from symptoms to the first available audiogram (years)	5.8± 7.7	Dyslipidemia	26 (37.1)
		Pulmonary disease ^Ꝭ^	2 (2.9)
Audiograms per patient	3.6± 1.3	Cardiac disease	4 (5.7)
		Epilepsy	1 (1.4)
Audiograms showing air-bone gap per patient	1.7± 1.3	Neurocognitive disease ^‡^	15 (21.4)
		Thyroid disease	9 (12.9)
Audiograms not showing air-bone gap per patient	1.9± 1.4	Previous chemotherapy	1 (1.4)
		Chronic headache	10 (14.3)
Ratio audiograms with air-bone gap/total number of audiograms for each patient	0.46± 0.33	Autoimmune disease	3 (4.3)
		Obstructive sleep apnea	6 (8.6)
Total audiograms (n = 252)			
Pure tone average (decibels)*†	37.8±9	Air-bone gap at 250 (AUDgap250/AUD)	116 (46)
Air-bone gap at 250 Hz†	8.7 ± 11.8	Air-bone gap at 500 (AUDgap500/AUD)	86 (34.1)
Air-bone gap at 500 Hz†	3.6±11.8	Air-bone gap at 1000 (AUDgap1000/AUD)	36 (14.3)
Air-bone gap at 1000 Hz†	1.5± 4.4	Air-bone gap at 2000 (AUDgap2000/AUD)	2 (0.8)
Air-bone gap at 2000 Hz†	0.04 ± 0.6		

Descriptive analysis of the LFABG

Among the 70 patients, 56 (80%) exhibited an LFABG at some point during the retrospective follow-up. Of the 252 total AUDs analyzed, 116 (46%) showed an LFABG in at least one tonal frequency. Notably, all 116 AUDs with LFABG demonstrated an LFABG at 250 Hz. However, this consistency did not extend to higher frequencies: 86 AUDs (34.1%) displayed LFABGs at 500 Hz, 36 AUDs (14.3%) at 1000 Hz, and only two AUDs (0.8%) at 2000 Hz. Table 2 presents the mean LFABG values for those AUDs exhibiting an LFABG, illustrating a pattern of decreasing incidence with increasing frequency.

**Table 2:** Audiograms showing the air-bone gap (n = 116)

Variable	N	Mean (± standard deviation)
Air-bone gap at 250 Hz	116	19 ± 10.4
Air-bone gap at 500 Hz	86	10.8 ± 7.6
Air-bone gap at 1000 Hz	36	10.4 ± 6.5
Air-bone gap at 2000 Hz	2	7.5 ± 2.5

Notably, the LFABG demonstrated considerable fluctuation. During the study period, of the patients who presented with an LFABG, 75.9% also exhibited AUDs characterized by pure low-tone SNHL without LFABGs in different evaluation periods.

Descriptive analysis of Ménière´s bouts and disease activity

Of the 70 patients included in the study, 58 (82.9%) exhibited unstable MD at some point during the retrospective follow-up period. To further quantify MDA, a ratio was calculated for each patient by dividing the number of evaluations coded as unstable by the total number of evaluations for that individual. The majority of patients demonstrated MDA within the range of 50-75% during the follow-up period (Table 3).

**Table 3:** Description of sample´s disease status (n = 70 patients) * Méniére´s disease evaluations with unstable disease divided by total number of evaluations (for each patient) x 100

Overall disease activity during follow-up *	Number of cases (frequency (%))
< 25 %	21 (30)
25-50 %	17 (24.3)
50-75 %	22 (31.4)
75-100 %	10 (14.3)

Association between the air-bone gap and Ménière's activity

In this sample, the coincidence index of unstable MD evaluations (UnsMDE) and the presence of LFABGs was 89.1% (Table 4), defined as the number of UnsMDE with a corresponding LFABG divided by the total number of UnsMDE. A chi-square test also revealed a significant association between the presence of LFABGs and UMDE (p < 0.001). Likewise, Spearman´s test showed a significant association between the number of UnsMDE and the number of AUDs with LFABGs (r = 0.796, p < 0.001). Age at first symptoms did not associate with LFABG incidence in the t-test (p = 0.443). Likewise, the time from symptoms to the first available audiogram did not associate with LFABG incidence (p = 0.384).

**Table 4:** Audiometric and clinical data matching: a descriptive analysis *Coincidence index: number of unstable disease evaluations with the corresponding LFABG/number of unstable disease evaluations LFABG: Low-frequency air-bone gap

Continuous variables	Mean (± standard deviation)	Categorical variables	Number of cases (frequency (%))
Patients (n = 70)		Audiogram and clinical evaluations (n = 252)	
Audiograms performed during clinically unstable disease	1.4±1	Audiograms performed during unstable disease	101(40.1)
Audiograms during unstable disease/ total number of audiograms	0.45±0.5	Audiograms with LFABG	116 (46)
		Evaluations coded as unstable MD and any air-bone gap	90 (35.7)
		Evaluations coded as unstable MD without air-bone gap	11 (4.4)
		Coincidence index*	90/101 (89.1)

**Figure 2:**
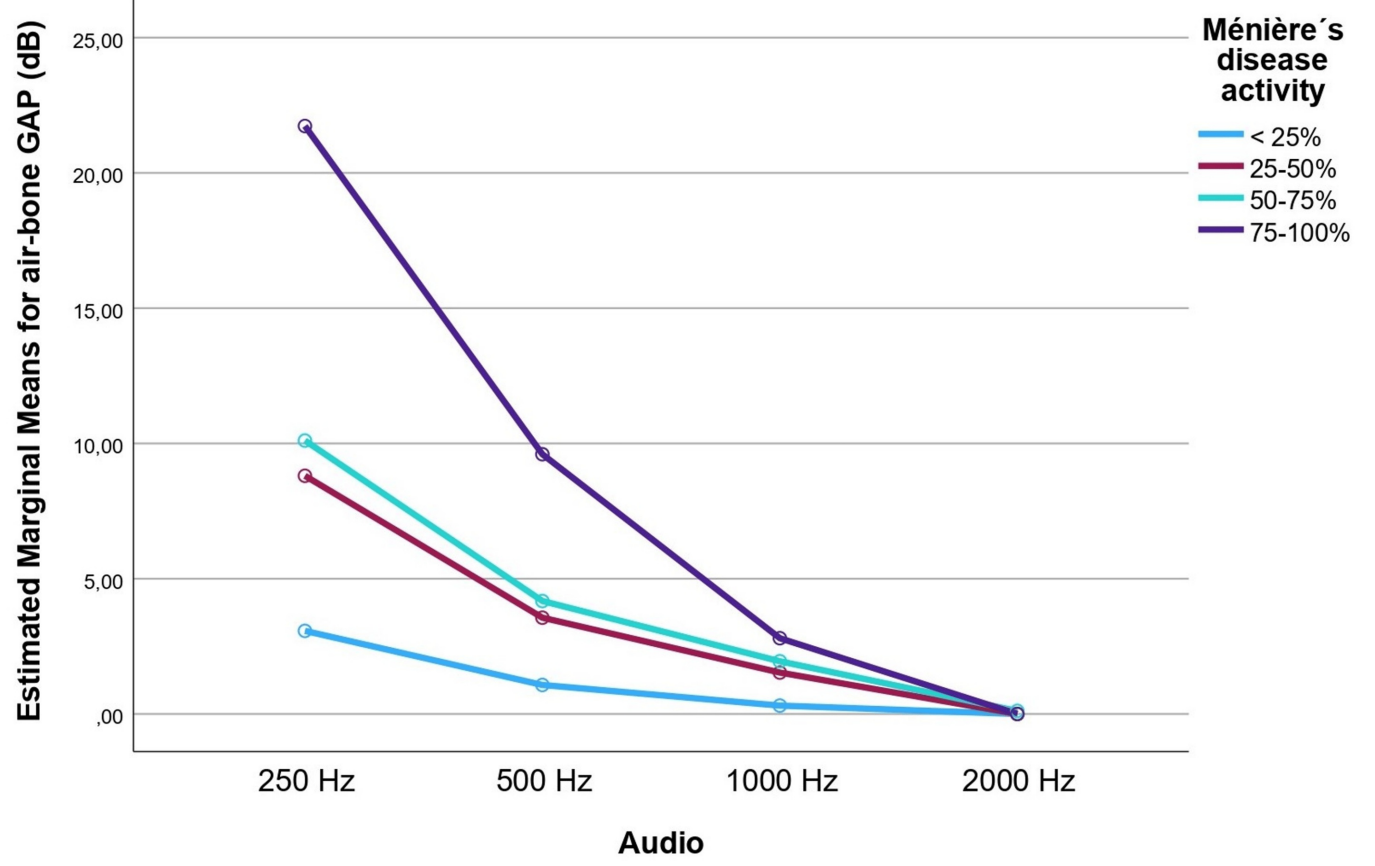
Mean low-frequency air-bone gap (LFABG) values (250-2000 Hz) across increasing levels of disease activity: <25%, 25-50%, 50-75%, and 100%. ANOVA results are presented for each frequency.

A two-way mixed ANOVA was performed to compare the effect of average LFABs at different tonal frequencies on MD´s activity (Figure 2). The assumption of sphericity was tested using Mauchly's test, which was significant, χ2(2) = 148, p <0 .001. Therefore, the degrees of freedom were corrected using the Greenhouse-Geisser method, ε = 0.438. There was a significant main effect of frequency and LFABG magnitude, F (1, 86.8) = 103.7, p < 0.001, η2 = 0.611. LFABGs were significantly higher on 250Hz (M = 9.3 ± 10) and 500 Hz (3.9 ± 5.3) than on 1000 Hz (M = 1.5 ± 3.4) and 2000 Hz (0.04 ± 0.3). This result defines the typical pattern of the ANTI-LAMBDA with decreasing air-bone gap values from 250 Hz through 2000 Hz.

There was a significant main effect of MDA on LFABG magnitude F (3,66) = 8.4, p < 0.001, η2 = 0.275. This means that MDA varied depending on the value of LFABGs. There was also a significant interaction between the frequency being tested and MDA on LFABG values F (3,66) = 12.3, p < 0.001, η2 = 0.359.

Post hoc comparisons were conducted using the Bonferroni correction. The difference between <25 % MDA and 75-100% MDA was statistically significant (mean difference: - 7.4, 95% CI [-11.5, -3.4], p < .001). Similarly, the difference between 25-50 % MDA and 75-100% MDA was statistically significant (mean difference: - 5.1, 95% CI [- 9.3, -0.85], p = 0.010). The difference between 50-75% MDA and 75-100 % MDA was also statistically significant (mean difference: - 4.5, 95% CI [-8.5, - 0.4], p = 0.023).

Sensitivity and specificity of air-bone gap toward unstable Ménière's disease

**Figure 3:**
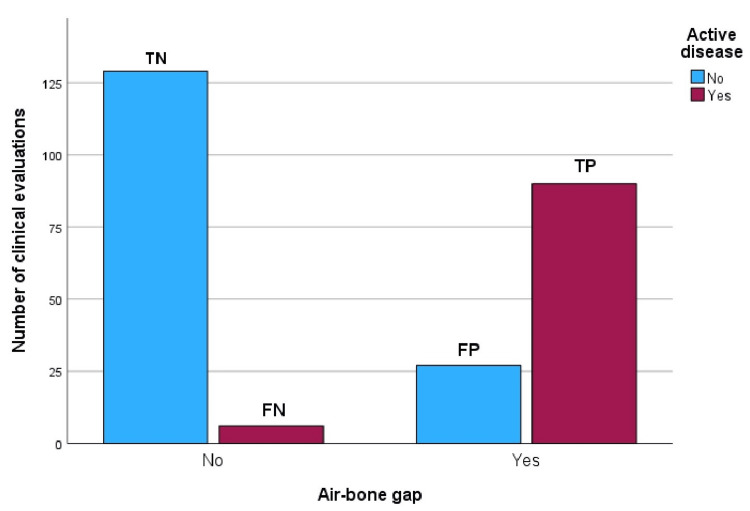
Bar chart demonstrating the classification of audiograms as true positive (TP), false positive (FP), true negative (TN), and false negative (FN) based on the presence of a low-frequency air-bone gap (LFABG) at 250 Hz and unstable Ménière's disease (MD), used to calculate sensitivity and specificity

After ascertaining the correlation between the LFABG and MDA, the sensitivity and specificity of the LFABG at 250 Hz for UnsMDE were calculated by a crosstabulation method. Sensitivity was 93.8 % and specificity 82.7 %. Figure 3 depicts negative and positive test results.

## Discussion

The ANTI-LAMBDA sign

The observation of LFABGs in MD, a phenomenon first described by Schuknecht in 1963 as "inner ear conductive/mixed hearing loss" [15], has since been sporadically reported in the literature. Our study not only confirms the frequent occurrence of LFABGs in MD (80% of patients) but also formally identifies and names a distinct pattern we have termed the "ANTI-LAMBDA" sign (A Non-statical Tonal Indicator - Low-frequency air-bone gap - of Ménière´s bouts and disease activity). This characteristic configuration, marked by a prominent LFABG at 250 Hz that progressively diminishes at higher frequencies (Figure 2), reflects inner ear conductive hearing loss.

**Figure 4:**
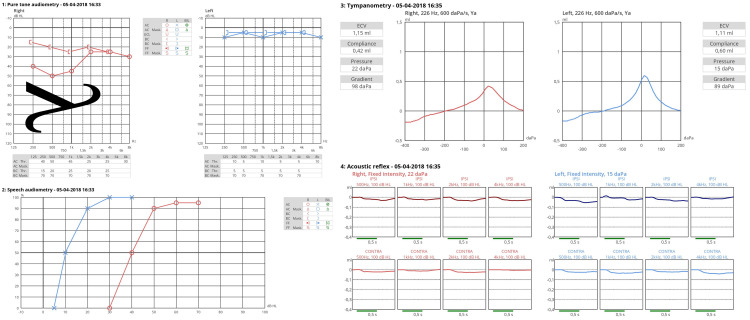
Audiogram depicting the ANTI-LAMBDA pattern during a Ménière´s disease bout. The pattern is indicative of inner ear conductive hearing loss and associates with increased disease activity. This characteristic pattern demonstrates a pronounced gap at 250 Hz and 500 Hz, with a progressive decrease in magnitude at higher frequencies, mirroring an inverted Greek lambda (Λ) symbol ANTI-LAMBDA: Non-statical Tonal Indicator Low-Frequency Air-Bone Gap of Ménière’s Bouts and Disease Activity

Our findings demonstrated a significant association between the ANTI-LAMBDA sign and clinically unstable MD, with LFABG magnitude correlating with disease activity, particularly at 250 Hz and 500 Hz. A significant positive correlation was observed between UnsMDE and AUDs exhibiting LFABG. A crosstabulation analysis revealed a high sensitivity (93.8%) and specificity (82.7%) for LFABG at 250 Hz in identifying UnsMDE, underscoring its potential as an indicative marker of MDA. This distinctive pattern, wherein an LFABG is consistently present at the lowest frequency (250 Hz) and progressively decreases at higher frequencies, has been termed the ANTI-LAMBDA sign due to its resemblance to an inverted Greek lambda (Λ) symbol. Figure 4 presents an illustrative case of the ANTI-LAMBDA sign in a patient with unstable MD.

The underlying pathophysiology of LFABGs in MD remains an area of active research, with two main hypotheses proposed: increased perilymphatic pressure dampening stapedial mobility, or direct pressure from a dilated saccule on the stapes footplate [10,14,16]. The latter hypothesis is supported by studies demonstrating the cochleocentric distribution of EH, with the saccule being particularly susceptible to pressure changes due to its high compliance [17]. Attyé et al. reported that the dilated saccule may directly contact the stapes footplate in up to 60% of MD cases, potentially hindering stapedial vibration and contributing to LFABG [18]. This mechanical interaction is further corroborated by findings of increased stapes annular ligament resonant frequency in MD patients by Gout et al. [19]. Recent research by Pai and Connor utilizing delayed gadolinium-enhanced MRI further substantiates the link between saccular distension and LFABG, suggesting that direct contact between the dilated saccule and the stapes footplate is a likely underlying mechanism [10]. EH originates in the cochlear duct and gradually progresses to the saccule through the ductus reuniens. This process culminates in saccular dilation, which may subsequently displace the stapes footplate, potentially serving as the underlying mechanism for the ANTI-LAMBDA sign [18]. As EH subsides, this process may reverse, potentially explaining the transient nature of the sign in many cases.

While the mechanical impact of EH appears to play a more significant role in the manifestation of MD symptoms compared to biochemical effects of membrane ruptures [20], the exact dynamics of saccular involvement remain unclear. Conflicting findings from Vestibular evoked myogenic potential (VEMP) studies, a neurophysiological tool for assessing saccular function, highlight the need for further research to elucidate the relationship between saccular function, LFABG, and MD´s activity [21-24]. Relevant studies observed differences in utricular and saccular function during acute MD attacks [21], with the saccule showing reduced function, potentially due to its anatomical proximity to bone [22]. This observation was further supported by studies showing more frequent abnormalities in cVEMPs (assessing saccular function) compared to oVEMPs (assessing utricular function) in MD patients [23]. Additionally, Angeli et al. [24] reported changes in cVEMP tuning across different stages of MD, suggesting dynamic shifts in saccular function over the course of the disease [24].

Our findings align with those of Yetiser et al. who described a similar pattern of "cochlear conductive hearing loss" in MD patients [12]. Conversely, Fraysse et al. also reported a correlation between vertigo frequency and saccular dilation, but did not find a significant relationship between the two parameters, contrasting with our observation of LFABG as a marker of unstable MD [20].

Notably, the ANTI-LAMBDA demonstrated fluctuation over time, with 75.9 % patients alternating AUDs characterized by pure low-tone SNHL with LFABG ones. This observation suggests that LFABG may be a dynamic and potentially reversible phenomenon in some MD patients, further emphasizing the need for longitudinal studies to track the evolution of this audiometric pattern.

The presence of LFABG in MD also raises questions regarding the stapedial reflex (SR). This study did not specifically assess this relationship due to inconsistent and incomplete documentation of SR in the retrospective patient records. While the elicitation of a SR in the presence of LFABG may seem counterintuitive, it is important to note that the footplate mobility is likely only dampened rather than fixed, potentially allowing for SR elicitation [11]. Alternatively, the presence of a third window effect, as proposed by some authors, may also contribute to LFABG and influence SR measurements [25].

The disappearance or reduction of LFABG over time, potentially due to vestibular atelectasis [26], aligns with the observation of reduced saccular function in advanced MD stages [27]. This could suggest that LFABG may be a more prominent feature in earlier stages of the disease. For that reason, we investigated whether the time elapsed between the onset of initial symptoms and the first AUD was associated with the presence of LFABG. However, our analysis revealed no significant correlation. This could be attributed to the relatively short average follow-up duration of less than six years from symptom onset in our cohort (Table 1), potentially limiting the ability to capture long-term evolution of LFABG in MD.

Strengths and limitations

This study represents the largest investigation to date on the association between a distinct pattern of LFABG and MDA. It is the first to identify this as a specific entity: the ANTI-LAMBDA. By exclusively including patients with unilateral MD, the authors ensured that the observed clinical manifestations and audiometric findings were attributable to the affected ear, minimizing potential confounding from bilateral disease.

However, this study has several limitations. The retrospective nature of the data collection may have introduced bias, as clinical records might not have consistently documented all relevant details. Additionally, while the study focused on audiometric assessment of LFABG, VEMPs and MRI with EH protocol were not assessed, which could have provided additional insights into saccular function, the relationship between the stapes footplate and the saccule, and the mechanical dynamics of LFABG. Moreover, the generalizability of the findings may be limited by the specific characteristics of the population and the diagnostic criteria employed.

## Conclusions

This study provides evidence for the association between LFABG, particularly the ANTI-LAMBDA sign, and unstable MD. Recognizing ANTI-LAMBDA as a potential marker of disease activity may have diagnostic and therapeutic implications in MD. In the particular case of bilateral MD, ANTI-LAMBDA could aid in identifying the affected ear and guiding interventions like intratympanic corticosteroid therapy. This article is intended to raise awareness of this phenomenon within the otolaryngology community. Future prospective studies incorporating VEMPs, SR, and MRI with EH protocol should validate these findings and investigate the ANTI-LAMBDA sign as a potential indicator of EH, even in patients who do not exhibit the full spectrum of MD.
